# Prise en charge des hypertendus dans la ville de Cotonou (Bénin) en 2011: connaissances attitudes et pratiques des médecins généralistes

**DOI:** 10.5830/CVJA-2015-094

**Published:** 2016

**Authors:** Martin Dèdonougbo Houenassi, Dokoui David, Léopold Houétondji Codjo, Angelo Cossi Attinsounon, Adebayo Alassani, Séraphin Ahoui, Albert Comlan Dovonou, Thierry Armel Adoukonou, Serge Hugues Mahougnon Dohou, Armand Wanvoegbe, Anthelme Agbodande

**Affiliations:** Health Unit, Education and Research in Cardiology, Faculty of Health, University of Abomey Calavi, Cotonou, Bénin; Health Unit, Education and Research in Cardiology, Faculty of Health, University of Abomey Calavi, Cotonou, Bénin; Department of Medicine and Medical Specialties, Faculty of Medicine, University of Parakou, Parakou, Bénin; Department of Medicine and Medical Specialties, Faculty of Medicine, University of Parakou, Parakou, Bénin; Department of Medicine and Medical Specialties, Faculty of Medicine, University of Parakou, Parakou, Bénin; Department of Medicine and Medical Specialties, Faculty of Medicine, University of Parakou, Parakou, Bénin; Department of Medicine and Medical Specialties, Faculty of Medicine, University of Parakou, Parakou, Bénin; Department of Medicine and Medical Specialties, Faculty of Medicine, University of Parakou, Parakou, Bénin; Department of Cardiology, Military Teaching Hospital, Parakou, Bénin; Department of Internal Medicine, National University Hubert Koutoukou Maga Hospital, Cotonou, Bénin; Department of Internal Medicine, National University Hubert Koutoukou Maga Hospital, Cotonou, Bénin

**Keywords:** prise en charge, hypertension artérielle, médecins généralistes, connaissances attitudes et pratiques, Afrique, management, arterial hypertension, general practitioners, knowledge, attitudes and practice, Africa

## Abstract

**But:**

Ce travail vise à évaluer les connaissances, attitudes et pratiques des médecins généralistes sur la prise en charge de l’hypertension artérielle à Cotonou.

**Méthodes:**

L’étude était transversale et descriptive basée sur une enquête multicentrique du 1er Mai 2011 au 31 Juillet 2011. Un recrutement de tous les médecins généralistes, volontaires exerçant dans les centres de santé privés, publics et confessionnels de la ville de Cotonou, ayant autorisé l’étude, a été fait. Le 7ème rapport de Joint National Committee (JNC7) a été utilisé comme référentiel pour l’évaluation de la prise en charge des hypertendus. Un auto-questionnaire adressé aux généralistes, testé et validé, a été utilisé pour recueillir ces données.

**Résultats:**

Au total, 41 médecins généralistes dans huit établissements sanitaires ont été inclus. Près de la moitié des généralistes (48.8%) ne connaissaient pas la définition de l’HTA. Seulement 25 généralistes (61.0%) pouvaient décrire les conditions de mesure de la pression artérielle. Dix généralistes (24.4%) étaient incapables de lister la moitié des examens du bilan minimum de l’hypertension artérielle (HTA). La majorité (92.7%) ne connaissait pas la notion de risque cardiovasculaire global. L’objectif tensionnel (TA ≤ 140/90 mmHg) n’était connu que par 18 (43.9%) médecins.

Les mesures hygiéno-diététiques seules (82.9%) et la monothérapie seule (70.7%) étaient les modalités thérapeutiques les plus prescrites. Les classes pharmacologiques antihypertensives prescrites étaient surtout les inhibiteurs calciques (82.9%), les inhibiteurs de l’enzyme de conversion (53.7%) et les diurétiques (36.6%). Les généralistes référaient les hypertendus aux cardiologues principalement pour non-maitrise du chiffres tensionnels (63,4%) et l’apparition de complications aigues (56.1%).

**Conclusion:**

Les connaissances des généralistes sur la prise en charge de l’HTA étaient insuffisantes et leurs attitudes et pratiques ne respectaient pas les recommandations internationales.

## Abstract (en)

**Objective:**

We aimed to assess the management of hypertensive patients by general practitioners in Cotonou city.

**Methods:**

This was a cross-sectional study based on a multicentre survey conducted from 1 May to 31 July 2011. We recruited all consenting general practitioners who worked in public and private centres in Cotonou city. We used the 7th report of the Joint National Committee to assess the management of hypertension by general practitioners. A tested and validated self-questionnaire was used to collect the data on hypertension management by general practitioners.

**Results:**

In eight centres that approved the study, 41 general practitioners were included. The definition of hypertension was known by 20 (48.8%) practitioners. Only 25 (61.0%) could describe the conditions for blood pressure measurement. Ten of them were unable to list half of the minimum recommended tests for hypertension, and the majority (92.7%) did not have any idea of global cardiovascular risk. The blood pressure goal was known by only 18 (43.9%) practitioners. Lifestyle (82.9%) and monotherapy (70.7%) were the therapeutic modalities most prescribed. Antihypertensive agents commonly used by practitioners were calcium channel blockers (82.9%), angiotensin converting enzyme inhibitors (53.7%) and diuretics (36.6%). The general practitioners referred their patients to cardiologists mainly for uncontrolled hypertension (63.4%) and the onset of acute complications (56.1%).

**Conclusion:**

The general practitioners’ knowledge of hypertension was insufficient and their management did not reflect international guidelines.

## But

L’hypertension artérielle (HTA), grave problème de santé publique, est en forte progression dans toutes les régions de l’Afrique sub-saharienne.^1^ Elle est un facteur de risque majeur dans la survenue d’accidents vasculaires cérébraux, d’insuffisance cardiaque, d’insuffisance rénale et de maladies coronaires qui représentent les principales causes de décès dans le monde.^2^,^3^ En dépit de la disponibilité d’une thérapie médicale efficace, plus de la moitié des hypertendus traités ont une pression artérielle mal contrôlée.^4^,^5^ Ce mauvais résultat est la conséquence d’une inefficacité du système de soins à tous les niveaux: dépistage, traitement, et observance thérapeutique.^6^ La mauvaise connaissance sur la prise en charge de l’HTA a été rapportée par Noubiap et al. au Cameroun en 2014. Peu de praticiens non cardiologues maitrisaient la définition de l’HTA, le bilan minimal, le choix des antihypertenseurs et la notion d’objectif thérapeutique.^7^ Safar et al. ont rapporté d’autres barrières à la gestion efficiente de l’hypertension artérielle. Il s’agissait entre autres de la faible fréquentation de ces centres par les patients, la mauvaise tenue des dossiers médicaux et le non respect des recommandations locales sur la prise en charge de l’HTA.^8^

Ce travail a été donc initié pour évaluer les connaissances, attitudes et pratiques des médecins généralistes sur la prise en charge de l’hypertension artérielle à Cotonou en 2011.

## Methodes

L’étude était multicentrique et avait inclus huit centres privés, publiques et confessionnels de médecine générale de la ville de Cotonou. Capitale économique du Bénin, Cotonou est une ville cosmopolite qui concentre l’essentiel des activités politiques, administratives, culturelles et sanitaire du Bénin.

Il s’agissait d’une étude transversale et descriptive menée du a1er Mai 2011 au 31 Juillet 2011. La population d’étude était composée de médecins généralistes, volontaires exerçant dans les différents centres de santé choisis depuis au moins le mois de janvier 2011.

Pour le choix des centres de santé, une demande d’autorisation a été adressée à l’administration de chaque établissementpratiquant la médecine générale. Seuls les centres qui ont donné leur autorisation ont été inclus. Dans ces centres le recrutement des médecins généralistes était systématique.

La variable dépendante était la prise en charge de l’hypertension artérielle par les médecins généralistes. Cette prise en charge a été jugée à partir des connaissances, attitudes et pratiques de ces derniers en matière d’hypertension artérielle. Le 7ème rapport de Joint National Committee (JNC7)9 a été utilisé comme référentiel pour l’évaluation de la prise en charge. Lestableaux 1tableaux 2 présentent les items abordés dans l’évaluation des connaissances, attitudes et pratiques des généralistes.

**Tableau 1:** Critères de jugement des connaissances des médecins généralistes en matière d’hypertension artérielle (HTA ) dans la ville de Cotonou (Bénin) en 2011

*Questions*	*Réponses attendues*
Quels sont les critères diagnostiques de l’HTA?	PAS ≥ 140 mmHg et/ou PAD ≥ 90 mmHg Mesurée chez un patient assis ou couché depuis au mois 05 minutes, les bras soutenus dans le plan du coeur. La caféine, l’exercice physique et le tabagisme doivent être évités au moins 30 minutes avant la mesure Valeurs confirmées au cours d’au moins trois consultations différentes (deux mesures à chaque consultation au cours d’une période de 3 à 6 mois) si toutes fois la gravité de l’HTA ne justifie pas un traitement antihypertenseur immédiat.
Quelle est la classification de l’HTA selon JNC 7 (Joint National Comittee 7)?	TA normale: TAS < 120 mmHg et TAD < 80 mmHg Pré-HTA: TAS entre 120–139 mmHg ou TAD entre 80–89 mmHg HTA grade I: TAS entre 140–159 mmHg ou TAD entre 90–99 mmHg HTA grade II: TAS ≥ 160 mmHg ou TAD ≥ 110 mmHg
Quel est le bilan minimum de l’HTA?	Biologie sanguine: glycémie à jeun, créatinine, taux d’hémoglobine, cholestérol (total, HDL), triglycérides, uricémie Biologie urinaire : protéinurie, hématurie Electrocardiogramme
Quels sont les autres facteurs de risque cardiovasculaire à rechercher chez l’hypertendu?	Age, sexe, antécédents familiaux d’accident vasculaire cérébral (AVC)/d’infarctus du myocarde(IDM) précoce ou de mort subite Tabac, diabète, dyslipidémie, sédentarité, obésité
Quels sont les critères de gravité de l’HTA	TAS ≥ 180 mmHg et/ou TAD ≥ 110 mmHg ou Diabète associé à l’HTA ou Syndrome métabolique (association de trois facteurs parmi l’HTA, l’hyperglycémie, l’hypocholestérolémie HDL, l’hypertriglycéridémie et l’obésité abdominale) ou Accumulation d’au moins trois facteurs de risque cardiovasculaire ou Existence d’une atteinte d’organe cible (hypertrophie ventriculaire gauche, protéinurie, rétinopathie hypertensive) ou de maladie cardiovasculaire (AVC, IDM, Artériopathie) ou de maladie rénale
Comment évaluer le risque cardiovasculaire absolu	Avec la table de Framingham ou autres scores A partir des valeurs tensionnelles, de cholestérols, des antécédents de tabagisme et de diabète.

**Tableau 2:** Critères de jugement l’attitude et pratique des médecins généralistes en matière d’hypertension artérielle (HTA ) dans la ville de Cotonou (Bénin) en 2011

*Questions*	*Réponses attendues*
Quelle(s) est (sont) la (les) mesure(s) hygiéno-diététique(s) de base que vous recommandez à tout hypertendu?	Restriction sodée; Arrêt du tabac; Modération de la consommation d’alcool; Réduction pondérale; Exercice physique régulier
Quels gestes d’examen physique faites-vous devant une HTA de découverte récente en absence de complications apparentes?	Recherche de contact lombaire, de souffle abdominal, de souffle cardiaque, des pouls; Calcul de l’IMC (après mesure de poids et de taille) Mesure du tour de taille Palpation de la thyroïde
Faut-il évaluer le risque cardiovasculaire global avant le traitement antihypertenseur?	Oui
Quelle(s) classe(s) d’antihypertenseur utilisezvous en première intention en monothérapie?	Diurétiques thiazidiques ou inhibiteurs calciques (IC) ou inhibiteurs de l’enzyme de conversion (IEC) ou antagonistes des récepteurs de l’angiotensine II (ARA II) ou bétabloquants (BB)
Quelle(s) stratégie(s) thérapeutique(s) adoptez-vous en première intension?	HTA grade I et risque faible: mesures hygiéno-diététiques (MHD) HTA et risque moyen: monothérapie ou bithérapie en combinaison fixe et MHD HTA et risque élevé: Au moins une bithérapie en combinaison fixe
Quelle(s) est (sont) le(s) association(s) d’antihypertenseurs à proscrire?	Deux antihypertenseurs de la même classe IEC et ARA II
Quels sont vos objectifs thérapeutiques au cours d’un traitement antihypertenseur?	Baisse TAS < 140 et TAD < 90 mmHg Prévention des accidents cardiovasculaire par la réduction du risque cardiovasculaire absolu
Quelle(s) est (sont) votre (vos) attitude(s) devant un patient hypertendu qui associe des tisanes aux antihypertenseurs?	Le sensibiliser sur le risque d’insuffisance rénale Arrêter les tisanes

Les médecins généralistes ont été investigués à l’aide d’un auto-questionnaire anonyme. L’enquête a été menée par un étudiant en 7ème année de médecine préalablement formé au questionnaire. Ce questionnaire a été testé sur 10 médecins généralistes et amélioré.

L’accord des autorités administratives des différents établissements sanitaire a été obtenu. Le consentement écrit des médecins généralistes enquêtés a été obtenu. La confidentialité des données recueillies sur les participants a été garantie. Desséances de restitution des résultats de cette enquête et une mise au point sur la gestion de l’hypertendu par le généraliste ont été organisés dans chaque structure sanitaire ayant participé à l’enquête.

## Traitement et analyse des données

Les données collectées ont été saisies, traitées et analysées avec le logiciel Epi info version 3.5.3. Les variables qualitatives étaient exprimées en pourcentage avec leur intervalle de confiance à 95% et les variables quantitatives en moyenne ± écart type.

## Resultats

L’enquête a été autorisée par huit établissements sanitaires. Il y avait trois centres publiques, trois centres privés et deux centres confessionnels. Les médecins généralistes présents et recensés étaient au nombre de 41. L’âge moyen des médecins était de 35.8 ± 9.6 ans avec les extrêmes de 24 à 66ans. Il y avait 28 (68.3%) hommes. En ce qui concerne l’expérience professionnelle, 20 (48.8%) médecins exerçaient depuis moins de cinq ans; 13 (31.7%) avaient une ancienneté entre 5 et 10 ans et 8 (19.5%) depuis au moins 10 ans.

## Connaissances des définition et classification de l’hypertension artérielle

Près de la moitié des généralistes (48.8%) ne connaissaient pas la définition exacte de l’HTA et 14 (34.1%) ne pouvaient pas donner les seuils de pression artérielle systolique (PAS) et de pression artérielle diastolique définissant l’HTA. Seulement 25 généralistes (61.0%) pouvaient décrire les conditions de mesure de la pression artérielle. Presque tous les généralistes (95.1%) ignoraient la classification de l’HTA selon JNC 7.

## Connaissances des evaluation de l’hypertendu

Concernant le bilan minimum de l’organisation mondiale de la santé (OMS), 10 généralistes (24.4%) étaient incapables de lister la moitié des examens constituant ce bilan. Un seul médecin (2.4%) a pu lister tous les examens de ce bilan. Le tableau 3 présente la proportion de médecins connaissant chacun des examens du bilan minimum de l’OMS.

**Table 3:** Proportion de médecins généralistes connaissant chacun des examens composant bilan minimum de l’Organisation Mondiale de la Santé à Cotonou (Bénin) en 2011

*Examens paracliniques*	*Effectif*	*Pourcentage*
Dans le sang		
Glycémie à jeun	33	80.5
Créatinine	35	85.4
Potassium	23	56.1
Cholestérol (total, HDL)	37	90.2
Triglycérides	34	82.9
Acide urique	12	29.3
Hémoglobine/hématocrite	09	21.9
Dans les urines (bandelette urinaire)		
Protéines, Hématies	03	07.3
Electrocardiogramme	33	80.5

Au moins 26.8% des médecins généralistes ne connaissaient aucun critère de gravité de l’HTA. Selon le facteur de gravité énuméré par ces médecins, on pouvait les répartir comme suit: (30) 73.2% pour la sévérité des chiffres tensionnels (TAS ≥ 180 mmHg ou TAD ≥ 110 mmHg), 25 (61%) pour l’existence d’atteinte d’organes cibles, 10 (24.4%) pour l’existence de complication cardiaque, 10 (24.4%) pour l’existence de maladie rénale, six (14.6%) pour l’accumulation d’au moins trois facteurs de risque cardiovasculaire et quatre (9.8%) pour l’existence d’un diabète associé. Aucun généraliste ne reconnaissant le syndrome métabolique comme critère de gravité de l’HTA. La majorité des généralistes (92.7%) ne connaissaient pas la notion de risque cardiovasculaire global.

## Connaissances des traitements de l’hypertension artérielle

Chez un hypertendu traité, l’objectif tensionnel (TA ≤ 140/90 mmHg) n’était connu que par 18 (43.9%) médecins. Encore moins de généralistes (17.1%) savaient que le traitement de l’HTA visait non seulement la baisse des chiffres tensionnels mais aussi la réduction du risque cardiovasculaire global et la prévention des accidents/maladies cardiovasculaires.

Un médecin généraliste sur 10 (9.8%) ne connaissait aucune mesure hygiéno-diététique spécifique à recommander à un hypertendu. La plupart des médecins savaient qu’il fallait recommander un régime pauvre en sel (90.2%), la consommation d’une alimentation pauvre en graisse (85.4%) et la pratique d’une activité physique régulière (85.4%). Par contre peu de médecins savaient que la réduction pondérale (4.9%), l’arrêt du tabac (29.3%) et la réduction de la consommation d’alcool (39%) faisant partie des conseils d’hygiène à donner à un hypertendu.

Les éléments de surveillance du traitement antihypertenseur cités par les médecins généralistes étaient les chiffres tensionnels (92.1%), la survenue de complications de l’HTA (34.1%) et les effets secondaires du traitement (15.6%).

## Évaluation des attitudes et pratiques des généralistes face à l’HTA

A l’examen physique de l’hypertendu, les gestes réalisés systématiquement par les médecins généralistes étaient la recherche d’anomalies auscultatoires cardiaques et vasculaires (90.2%), la palpation des pouls périphériques (39%) et le calcul de l’indice de masse corporelle (7.9%). Aucun médecin ne mesurait le tour de taille des patients. Aucun médecin généraliste n’évaluait le niveau de cardiovasculaire global des patients avant la mise sous traitement antihypertenseur.

En ce qui concerne les modalités thérapeutiques fréquemment prescrites par les médecins, on retrouve les mesures hygiénodiététiques (MHD) seules dans 82.9%, la monothérapie seule dans 70.7%, la bithérapie seule dans 14.6%, l’association MHD-monothérapie dans 53.7% et l’association MHD-bithérapie dans 14.6% des cas. Aucun médecin ne prescrivait la trithérapie antihypertensive. Les classes pharmacologiques antihypertensives prescrites étaient les inhibiteurs calciques (82.9%), les inhibiteurs de l’enzyme de conversion (53.7%), les diurétiques (36.6%), les bétabloquants (14.6%) et les antihypertenseurs centraux (7.3%).

Lors du suivi des hypertendus, les motifs pour lesquels les généralistes référaient les patients aux cardiologues étaient la non-maitrise des chiffres tensionnels (63.4%), l’apparition de complications aigues (56.1%) et la référence systématique de tous les cas d’HTA (36.6%).

## Discussion

Ce travail nous a permis de décrire les connaissances, attitudes et pratiques des médecins généralistes de la ville de Cotonou sur la prise en charge de l’HTA. La prise en compte des établissements sanitaires aussi bien publiques, confessionnels que privés et le recrutement systématique de tous les médecins généralistes y exerçant nous ont permis de réduire les biais de sélection. Cependant, le choix non aléatoire de ces établissements empêchera la généralisation de nos résultats à toute la ville de Cotonou. L’usage d’un auto-questionnaire adressé aux médecins garantit la sincérité des déclarations recueillies.

Au terme de cette étude, nous avons observé que les médecins généralistes ne maitrisaient pas la définition de l’HTA ni l’évaluation de l’hypertendu conformément aux recommandations de JNC7. Les objectifs tensionnels n’étaient pas connus de tous les médecins généralistes de même la prescription des MHD. Les médicaments anti hypertenseurs recommandés sont bien connus par les généralistes mais les diurétiques thiazidiques sont peu prescrits par ces derniers.

La mauvaise connaissance des généralistes sur l’HTA n’est pas spécifique à la ville de Cotonou. En effet, au Pakistan en 2010, Rehman et al. ont rapporté que 25.5% des médecins généralistes ne connaissaient pas la valeur seuil qui définit l’HTA.^10^ À l’ouest du Cameroun en 2012, 36.4% des médecins généralistes utilisaient de fausses valeurs pour définir l’HTA.^7^ La situation est encore pire au Burkina Faso où, en 2002, 65% des médecins avaient basé la définition de l’HTA essentiellement surla pression artérielle systolique.^11^ De même, en Chine, Chen et al., en 2011, ont observé une mauvaise définition de l’HTA par les généralistes dans 55.8%.^12^

Pour l’évaluation de l’hypertendu, un bilan minimum est recommandé en complément de l’examen clinique et a pour but de rechercher les facteurs de risque associés à l’HTA, les atteintes des organes cibles et co-morbidités de l’HTA (insuffisance rénale, insuffisance cardiaque, coronaropathie) et les signes orientant vers une éventuelle hypertension artérielle secondaire (hypokaliémie, insuffisance rénale, anémie).^9^,^13^,^14^ Dans notre série, 80% des médecins généralistes demandaient le bilan minimum mais il n’était à moitié conforme que chez 24.4% des praticiens. Notre observation rejoint celle de Noubiap et al. qui avaient rapporté une demande de bilan minimum par 87% des généralistes avec un taux d’adéquation de faible (10.4%).^7^

Cette déficience observée dans l’évaluation de l’hypertendu nous permet de comprendre la méconnaissance des critères de gravité de l’HTA par les généralistes. Si la sévérité des chiffres tensionnels (≥ 180/110 mmHg) et les retentissements viscéraux étaient connus comme éléments de gravité de l’HTA par certains généralistes dans notre série, aucun des ces derniers ne considérait le syndrome métabolique, et seulement 16.9% de ces derniers considéraient le diabète comme critères de haut risque cardiovasculaire. De même, aucun généraliste ne connaissait la notion du risque cardiovasculaire global (RCVG). Pourtant, l’évaluation du RCVG est une étape majeure de la prise en charge de l’hypertendu. En effet, le niveau de risque découle du bilan de l’hypertendu et oriente le traitement médicamenteux de même que la définition des objectifs tensionnels.^9^,^13^,^14^ La méconnaissance du RCVG par les praticiens expose les patients à une mauvaise prise en charge avec comme conséquence un mauvais contrôle tensionnel et un risque accru d’accident cardiovasculaire.^15^

Sur le plan thérapeutique l’objectif tensionnel n’était connu que par 43.9% des généralistes et seulement 14.1% tenaient compte du RCVG pour définir le traitement de leurs patients hypertendus. Au Pakistan en 2005, Jafar et al. avaient observé que 52.3% des généralistes connaissaient les objectifs tensionnels.^16^ Ce taux était de 44.2% au Cameroun en 2014.^7^ La place des mesures hygiénodiététiques (MHD) dans le traitement de l’HTA était bien connue par la plus part des médecins généralistes dans notre étude (90.2%) de même que celle de Chen et al. (99.3%).^12^ Les MHD sont moins pratiquées dans d’autres séries africaines: 77.9% au Cameroun^7^ et 50% en Afrique du Sud.^17^ Il est important de reconnaitre cependant que, même si ces MHD ont été souvent prescrites par ces généralistes, les différentes composantes de ces MHD n’étaient pas toujours bien maitrisées par ces praticiens. Par exemple, nous avons observé dans notre enquête que peu des médecins savaient que l’arrêt du tabac (29.3%), la réduction de la consommation d’alcool (39%) et la réduction pondérale (4.9%) faisaient partie des MHD adressées aux hypertendus.

Selon les travaux de Chen et al., quatre problèmes principaux entravaient la prescription des MHD par les généralistes: la mauvaise observance des patients, le manque de temps de consultation à consacrer à l’éducation sur les MHD, le manque de compétence du médecin en matière de MHD et le manque de technique pour enseigner ces MHD.^12^ Les classes pharmacologiques les plus prescrites en monothérapie dans notre étude étaient les inhibiteurs calciques suivis des inhibiteurs de l’enzyme de conversion, les diurétiques thiazidiques et les bétabloquants. Au Cameroun, c’étaient les diurétiques de l’anse qui étaient les plus prescrits (49.3%).^7^ Au Pakistan, par contre, on a observé curieusement une forte prescription d’anxiolytiques comme médication de première intention contre l’HTA.^6^ Actuellement les classes d’antihypertenseurs recommandées enpremière intention par les sociétés savantes restent les diurétiques thiazidiques, les inhibiteurs calciques, les inhibiteurs de l’enzyme de conversion, les antagonistes des récepteurs de l’angiotensine II et les bétabloquants.^9^,^13^,^14^ Il existe une préférence pour les inhibiteurs calciques et les thiazidiques chez les sujets de race noire.^18^

L’étude ALLAT a montré que les thiazidiques confèrent les mêmes bénéfices que les autres classes thérapeutiques recommandées mais ils sont supérieurs aux bétabloquants, aux inhibiteurs calciques et aux inhibiteurs de l’enzyme de conversion dans la prévention des accidents vasculaires cérébraux et de l’insuffisance cardiaque.^19^ Ces bénéfices pronostiques s’ajoutent au faible coût des thiazidiques. L’accent devrait donc être mis sur la prescription des thiazidiques dans nos pays à faible revenu. Bien entendu, il faudra tenir compte de leurs effets secondaires métaboliques et les prescrire à faible dose et surtout en association avec d’autres classes d’antihypertenseurs, notamment les inhibiteurs calciques et les IEC.

L’ensemble de ces données de la littérature sur la prise en charge de l’HTA par les généralistes doit être considérée comme une alerte. Une riposte à cette situation désastreuse s’impose. Elle doit consister en une formation universitaire et post universitaire. Une priorité doit être donnée aux maladies non transmissibles et particulièrement l’HTA dans les curricula de formation des médecins généralistes dans nos pays. De même les autorités politico-administratives et l’ordre des médecins devraient soutenir les formations post universitaires des médecins praticiens. Ce besoin de formation a été clairement exprimé par les généralistes dans l’enquête de Chen et al. en Chine en 2011.^12^ La négligence de cette incapacité des généralistes à gérer l’HTA contribuera forcement à un mauvais contrôle de cette pathologie comme l’ont démontré quelques travaux.^16^,^20^-^22^

## Conclusion

La connaissance des généralistes sur la prise en charge de l’HTA était insuffisante et leur gestion ne respectait pas les recommandations internationales.
